# Comparison Between Intravenous and Intramuscular Octreotide in the Management of Heyde’s Syndrome

**DOI:** 10.7759/cureus.25461

**Published:** 2022-05-29

**Authors:** Hira I Cheema, Russell Roark, Seemeen Hassan, Kalyan Chakrala, Benjamin Tharian, Jiannis Anastasiou

**Affiliations:** 1 Internal Medicine, Baptist Health Medical Center, Little Rock, USA; 2 Gastroenterology, University of Arkansas for Medical Sciences, Little Rock, USA; 3 Gastroenterology, Medical Center Hospital, Odessa, USA

**Keywords:** aortic valve replacement, endoscopy, anemia, von willebrand factor, aortic stenosis, octreotide, heyde’s syndrome

## Abstract

Heyde’s syndrome is defined as a triad of aortic stenosis, anemia due to angiodysplasia-related bleeding, and von Willebrand syndrome type 2A. It is a rare disease and a diagnostic challenge. Treatment modalities include symptomatic management, blood transfusions, aortic valve replacement, and medications such as octreotide. Here, we report the case of a patient who was resistant to symptomatic management, aortic valve replacement, as well as intravenous octreotide.

## Introduction

American internist Edward C. Heyde originally described in a letter to the New England Journal of Medicine (NEJM) a pattern of symptoms in patients who had aortic stenosis with bleeding angiodysplasia [1]. The mechanism by which aortic stenosis leads to the formation of angiodysplasia is through the development of an acquired form of von Willebrand disease (vWF) [2,3].

Under normal blood flow, vWF remains in its coiled form until it is exposed to damaged endothelium and binds to collagen. This causes the protein to uncoil and allows for aggregation and binding of platelets to help form clots. In aortic stenosis, the turbulent flow of blood through the stenosed aortic valve leads to mechanical disruption of von Willebrand multimers [2,4,5]. When vWF uncoils while passing through the stenotic aortic valve, it is cleaved by the enzyme ADAMTS13. This cleaved form cannot bind to damaged collagen and prevents it from forming clots. Hence, patients who have symptomatic bleeding and undergo an aortic valve replacement tend to clinically improve [6,7].

There are pharmacologics that can aid in treating the syndrome. Octreotide is a commonly used medication. There are different mechanisms by which octreotide can reduce and inhibit gastrointestinal (GI) bleeding. Of note are inhibition of hormones (pepsin and gastrin) and acid secretion, increased platelet aggregation, decreased duodenal and splanchnic blood flow, increased vascular resistance, and inhibited angiogenesis [8-12]. The use of octreotide in the treatment of angiodysplasia has been reported in case reports, a small series, and a meta-analysis [13-20]. These studies separately report patients who received either subcutaneous octreotide or once-monthly intramuscular octreotide [18,20].

## Case presentation

**Figure 1:**
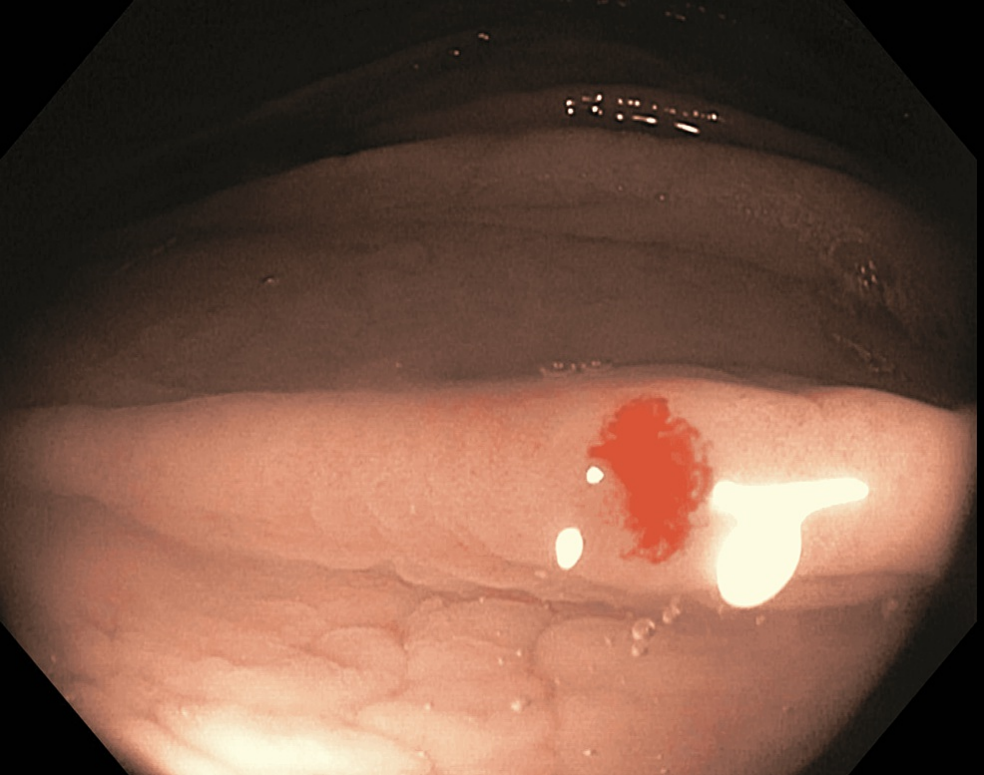
AVM seen in the first part of the duodenum during EGD. AVM: arteriovenous malformation; EGD: esophagogastroduodenoscopy

We report the case of a 76-year-old male with a medical history of hypertension and carotid artery stenosis. He was first referred for evaluation of multiple episodes of anemia secondary to GI bleeding that required monthly blood transfusions. At the time of referral, the patient denied abdominal pain, active bleeding, usage of aspirin (ASA), or non-steroidal anti-inflammatory drugs (NSAIDs). The patient had stopped taking warfarin three months prior, which was intended for the management of carotid artery stenosis. Esophagogastroduodenoscopy (EGD) and colonoscopy done two weeks prior to the time of referral were negative. Laboratory work showed a hemoglobin (Hb) of 8.7 g/dL and platelets of 237,000/µL. Physical examination was significant for skin pallor and a systolic murmur. The patient underwent elective EGD again and was found to have several actively bleeding arteriovenous malformations (AVMs) in the stomach and the first part of the duodenum (Figure 1).

**Figure 2:**
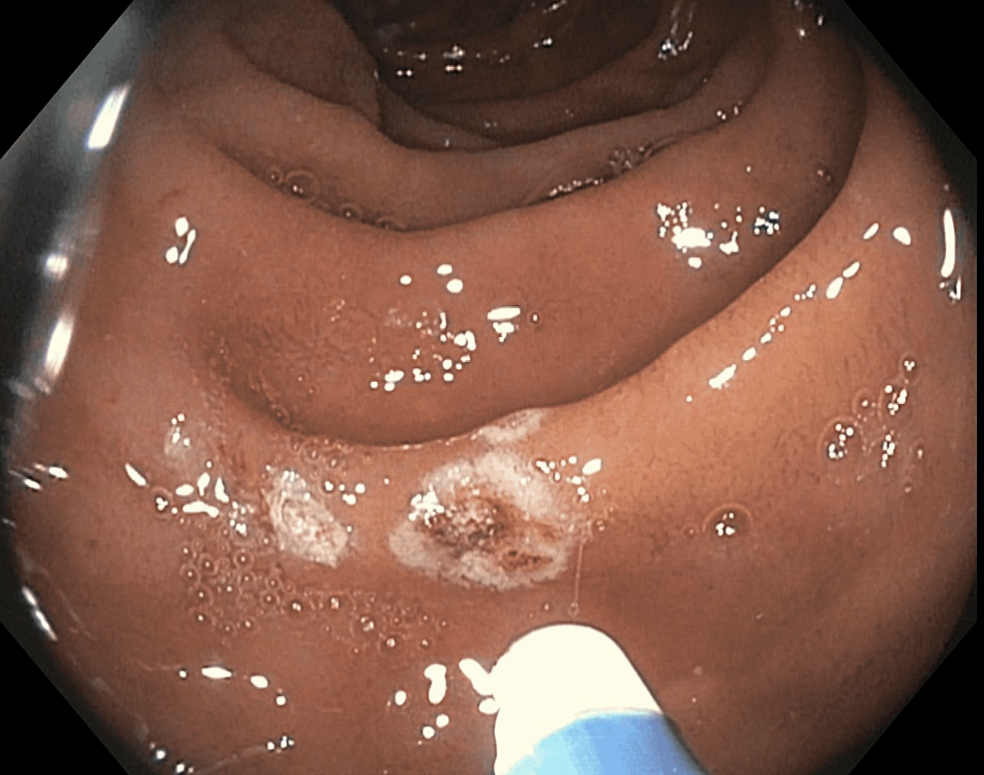
APC for duodenal AVMs. APC probe is seen. Duodenal AVM is coagulated. APC: argon plasma coagulation; AVM: arteriovenous malformation

The bleeding AVMs were successfully treated with argon plasma coagulation (APC) (Figure 2).

The patient was discharged to follow up as an outpatient. A month later, he was admitted to the hospital due to worsening shortness of breath with reports of melena for the past week. He had a repeat Hb drawn that was 8.0 g/dL. Repeat EGD showed bleeding angiodysplasias in the duodenum, which were again coagulated with APC. A decision was made to start the patient on intravenous (IV) octreotide 50 mg twice a day as an outpatient.

The patient presented to the hospital one month later for worsening shortness of breath and hypoxia. At the time of admission, the patient had been on IV octreotide for two weeks and complained about ongoing GI bleeding with melena. Labs showed Hb of 6.7 g/dL. Echocardiogram showed critical aortic stenosis with a valve area of 0.92 cm^2^. The patient subsequently underwent bioprosthetic valve replacement. He also received two drug-eluting stents for coronary artery disease and was started on antiplatelet therapy with clopidogrel.

One month after the bioprosthetic valve replacement, the patient presented again to the hospital with weakness and was found to be anemic with Hb of 5.5 g/dL. The patient had been receiving blood transfusions every two weeks for eight months due to chronic anemia. Repeat EGD again showed multiple AVMs in the duodenum which were controlled after APC.

At this time, he was started on octreotide 20 mg intramuscular (IM) injection monthly as previous treatment with IV octreotide had not shown much improvement in symptoms (Hb of 6.6 g/dL). Over the course of nine months during follow-ups, the patient reported steady improvement their energy and requiring less blood transfusions. From March to July, his Hb improved from 6.6 to 10.7 g/dL. The octreotide dose was increased to 30 mg at this point. In the next five months, his Hb improved steadily from 10 to 12 g/dL with minimal to no blood in stools.

## Discussion

We are reporting for the first time the comparison between IV and IM octreotide in the resolution of GI bleeding due to angiodysplasia in patients with aortic stenosis. Our patient received IV octreotide 50 mg twice a day for two months without significant relief in symptoms. Even after aortic valve replacement, he had minimal resolution of bleeding on IV octreotide. We then started therapy with IM octreotide with close follow-up as an outpatient. For the first five months on 20 mg daily octreotide, the patient did report some GI bleeding and required monthly blood transfusions. However, after increasing the dose to 30 mg IM per month, his bleeding eventually resolved and he maintained a steady Hb from 10 to 12 g/dL after four months of follow-up. There are no reported studies comparing the difference between the efficacy of IV and IM octreotide. This case suggests that there may be different efficacy of octreotide depending on the route of administration, and new studies comparing the difference between IV and IM octreotide may aid in improving overall care in patients with Heyde’s syndrome as well as other angiodysplasia syndromes.

## Conclusions

Although a single case, our report highlights an important aspect of treatment for Heyde’s syndrome. If untreated or underdiagnosed, it can become a debilitating condition. IM octreotide is an easy-to-manage treatment regimen. Our patient reported complete resolution of symptoms during subsequent follow-ups. Further reports on the differences in patient outcomes can help improve treatment modalities and patient care.
